# Low serum chloride concentration and the in-hospital mortality of patients with acute decompensated heart failure: a meta-analysis

**DOI:** 10.3389/fmed.2026.1826977

**Published:** 2026-07-10

**Authors:** Jiaqin Wang, Xianrong Li

**Affiliations:** 1 Department of Cardiology, CCU, West China Hospital, Sichuan University/West China School of Nursing, Sichuan University, Chengdu, China; 2 Department of Urology, The Second People’s Hospital of Yibin, Yibin, China

**Keywords:** acute decompensated heart failure, hypochloremia, meta-analysis, mortality, serum chloride

## Abstract

**Background:**

Hypochloremia is increasingly recognized as a marker of neurohormonal activation and adverse prognosis in acute decompensated heart failure (ADHF). However, the magnitude and consistency of its association with short-term mortality remain uncertain. This meta-analysis aimed to evaluate the relationship between admission serum chloride levels and in-hospital mortality among patients hospitalized with ADHF.

**Methods:**

PubMed, Embase, and Web of Science were searched for cohort studies reporting the association between admission serum chloride levels and in-hospital mortality in ADHF. Pooled risk ratios (RRs) with 95% confidence intervals (CIs) were calculated using a random-effects model accounting for the possible influence of heterogeneity.

**Results:**

Seven studies comprising eight independent cohorts and 17,239 ADHF patients were included. Overall, 2,422 (14.0%) had admission hypochloremia, and 1,242 (7.2%) died during hospitalization. Hypochloremia was associated with significantly higher in-hospital mortality (RR: 2.24, 95% CI: 1.60–3.13, *p* < 0.001; *I*^2^ = 69%). Subgroup analyses according to study design, mean ages of the patients, or the proportions of men did not significantly affect the results (*p* for subgroup difference all > 0.05). A stronger association was observed when chloride was measured at admission (RR: 2.62) compared with within 24 hours (RR: 1.46, *p* for subgroup difference < 0.001). Associations were similar across chloride cutoffs (<98, <96, <95 mmol/L) and analytic strategies (univariate vs. multivariate, *p* for subgroup difference all > 0.05).

**Conclusion:**

Admission hypochloremia is associated with higher in-hospital mortality in patients with ADHF.

**Systematic review registration:**

https://www.crd.york.ac.uk/prospero/, identifier registration number CRD420251275904.

## Introduction

Acute decompensated heart failure (ADHF) is a leading cause of emergency cardiovascular hospitalization worldwide and accounts for substantial short-term morbidity, mortality, and healthcare expenditure (1, 2). Patients admitted with ADHF face an in-hospital mortality rate of approximately 4–10%, with risk further amplified by older age, comorbidities, hemodynamic instability, and multi-organ dysfunction (3, 4). Although contemporary management—including loop diuretics, vasodilators, noninvasive ventilation, and guideline-directed medical therapy optimization—has modestly improved symptoms, short-term prognosis remains poor, and early death continues to occur largely due to congestion, hypoperfusion, arrhythmic events, and progressive metabolic and neurohormonal dysregulation (3, 4). In this context, identifying simple, readily available biomarkers that can enhance early risk stratification is of critical clinical importance, as they may guide the intensity of monitoring, decongestion strategies, and therapeutic decision-making during the vulnerable early phase of hospitalization (5).

Chloride is the most abundant extracellular anion and plays essential roles in maintaining acid–base balance, neurohormonal signaling, renal salt–water regulation, and responsiveness to diuretic therapy (6, 7). Experimental and clinical studies have demonstrated that low serum chloride can activate the renin–angiotensin–aldosterone (RAAS) system, augment vasoconstriction, impair natriuresis, and promote persistent congestion—pathophysiologic pathways that are particularly relevant in ADHF (8–11). Hypochloremia may also reflect aggressive loop diuretic exposure, dilutional states, metabolic alkalosis, or impaired tubular chloride reabsorption, each contributing to hemodynamic compromise (12, 13). Several cohort studies have reported that lower admission chloride levels are associated with worse outcomes in patients with heart failure (HF), and a previous meta-analysis focusing mainly on long-term outcomes showed that hypochloremia predicted increased all-cause mortality up to 1–2 years after an ADHF admission (14). However, the prognostic significance of hypochloremia for acute mortality—particularly in-hospital mortality—remains less well defined. Individual ADHF studies have yielded inconsistent findings, likely due to variations in chloride cutoffs, timing of measurement, population characteristics, and analytic approaches (15–21). Given the clinical relevance of early mortality in ADHF and the availability of new cohort data, a rigorous synthesis is warranted. Therefore, we conducted a meta-analysis to evaluate the association between admission hypochloremia and in-hospital mortality among patients hospitalized with ADHF.

## Methods

The conduct and reporting of this meta-analysis adhered to the PRISMA 2020 recommendations (22) and relevant guidance from the Cochrane Handbook (23), encompassing protocol development, data collection, statistical synthesis, and presentation of findings. The protocol was preregistered with PROSPERO (CRD420251275904).

### Database search

Eligible studies were located through an extensive literature search of PubMed, Embase, and Web of Science, employing a broad set of predefined search terms, which included: (1) “chloride” OR “chloride level” OR “serum chloride” OR “hypochloremia” OR “hypochloraemia”); (2) “acute decompensated heart failure” OR “ADHF” OR “acute heart failure” OR “acute HF” OR “decompensated heart failure” OR “decompensated HF” OR “heart failure”; and (3) “mortality” OR “death” OR “deaths” OR “survival” OR “all-cause mortality” OR “overall survival.” The search was restricted to human research and full-text articles published in English in peer-reviewed journals, and grey literature sources, conference proceedings, clinical trial registries, and unpublished studies were not systematically searched. To supplement the electronic search, reference lists of pertinent original studies and reviews were manually examined to identify additional eligible publications. All databases were searched from their inception through October 29, 2025. Detailed search strategies for each database are provided in Supplementary File 1.

### Study inclusion and exclusion

Study eligibility was defined according to the PICOS framework:

P (Population): Patients diagnosed with ADHF on hospital admission, with the diagnosis of ADHF consistent with the criteria used in the original studies.

I (Exposure/Index): Serum chloride level measured at baseline (on admission or within 24 h of admission), analyzed as categorical variable (low vs. normal chloride). The cutoff for defining a low serum chloride was also consistent with that used in original studies. Patients with a low serum chloride level at baseline were considered as exposure.

C (Comparator): Patients with higher or normal admission serum chloride levels at baseline served as controls.

O (Outcomes): Inhospital mortality, compared between patients with and without hypochloremia at baseline.

S (Study Design): Longitudinal observational studies, including: Prospective cohort studies; Retrospective cohort studies; Follow-up analyses from registries/databases; *Post-hoc* analysis of clinical trials; Nested case-control studies; Studies with at least one follow-up mortality endpoint. Full-text articles published in peer-reviewed journals.

We excluded cross-sectional studies, case-control studies without longitudinal follow-up, case series, case reports, reviews, editorials, conference abstracts, unpublished data, and animal or laboratory studies. Studies focusing exclusively on chronic heart failure without defining acute decompensation were also excluded. Research that did not evaluate serum chloride on admission, did not report mortality outcomes, or lacks sufficient information to derive effect estimates were also excluded. If multiple publications use overlapping cohorts, the one with the largest sample size was included to avoid duplication.

### Study quality evaluation

Two reviewers independently performed the literature search, study screening, quality appraisal, and data extraction, with any disagreements resolved through between the two authors. Study quality was assessed using the Newcastle–Ottawa Scale (NOS) (24), which evaluates selection, adjustment for confounding, and outcome assessment, yielding scores from 1 to 9, with higher scores indicating better methodological rigor. Studies scoring ≥7 were classified as high quality.

### Data collection

Extracted data included study-level information (author, publication year, country, and study design), participant characteristics [diagnosis, number of patients included, mean age, sex distribution, proportions of patients with heart failure with reduced ejection fraction (HFrEF), and proportions of patients with ischemic etiology], exposure characteristics (detailed timing of serum chloride measurement, cutoff values for defining hypochloremia, and number of patients with hypochloremia at admission in each study), numbers of patients who died during hospitalization, and covariates adjusted for in the estimation of the association hypochloremia at admission and inhospital mortality of patients with ADHF.

### Statistical analysis

The association between hypochloremia at admission and inhospital mortality of patients with ADHF was summarized using risk ratios (RRs) with corresponding 95% confidence intervals (CIs). When necessary, RRs and standard errors were derived from reported CIs or *p*-values and subsequently log-transformed to stabilize variance and approximate normality (23). Between-study heterogeneity was examined using the Cochrane Q statistic and the *I*^2^ metric (25), with thresholds of <25, 25–75, and >75% interpreted as low, moderate, and high heterogeneity, respectively. Pooled estimates were generated using a random-effects model to account for underlying variability across studies (23). Robustness of the overall effect was evaluated through leave-one-out sensitivity analyses by excluding one study at a time (26). We conducted prespecified subgroup analyses to explore whether study characteristics influenced the observed associations. Stratifications included study design (prospective vs. retrospective), mean ages of the patients, proportions of men, timing of serum chloride measurement, cutoffs for defining hypochloremia, and analytic models used (univariate vs. multivariate analysis). Potential publication bias was evaluated using funnel plot symmetry, visual inspection, and Egger’s regression test (27). A two-sided *p* < 0.05 was considered statistically significant. All analyses were performed using RevMan (version 5.3; Cochrane Collaboration, Oxford, United Kingdom) and Stata (version 17.0; StataCorp, College Station, TX, United States).

### Certainty of evidence

The certainty of evidence for the primary outcome was evaluated using the Grading of Recommendations Assessment, Development and Evaluation (GRADE) framework (28). The assessment considered risk of bias, inconsistency, indirectness, imprecision, and publication bias. The certainty of evidence was categorized as high, moderate, low, or very low.

## Results

### Study inclusion

**FIGURE 1:**
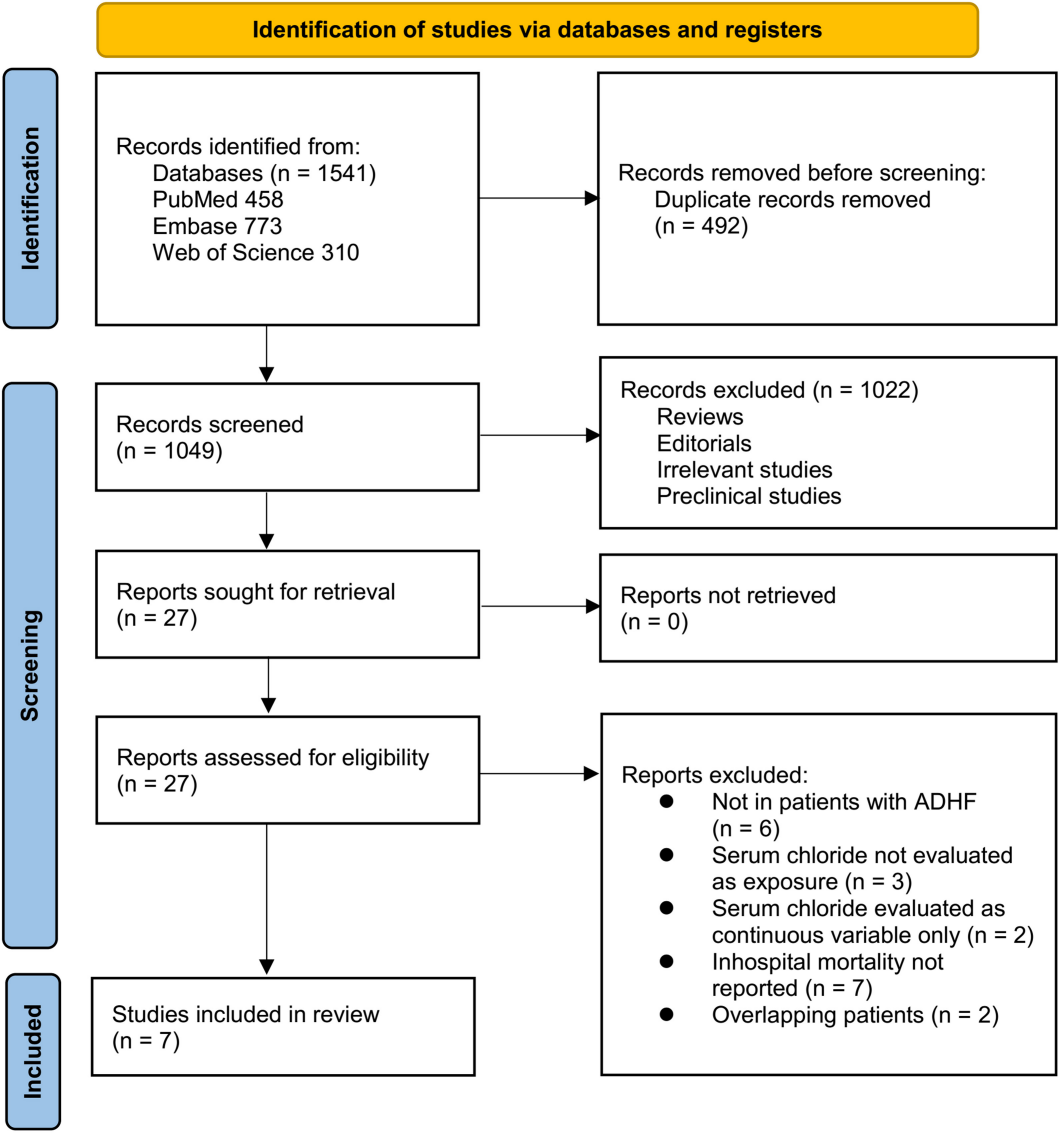
Flowchart of database search and study inclusion.

Figure 1 depicts the study selection workflow. A total of 1,541 records were retrieved from the three databases, of which 492 duplicates were removed. Screening of titles and abstracts resulted in the exclusion of 1,022 records that did not meet the eligibility criteria. The full texts of the remaining 27 articles were evaluated independently by two reviewers, and 20 were excluded for reasons shown in Figure 1. Ultimately, seven studies met all criteria and were included in the quantitative synthesis (15–21).

### Overview of the study characteristics

The key characteristics of the included studies are summarized in Table 1. Because one study included two independent cohorts (16), these cohorts were included in the meta-analysis separately, making eight cohorts available for the meta-analysis. These studies were published between 2016 and 2025, and were conducted across Croatia, Japan, China, Ethiopia, the United States, Spain, and Russia. Collectively, these studies enrolled 17,239 patients hospitalized with ADHF. Study designs included four prospective cohorts (15–17, 20) and four retrospective cohorts (16, 18, 19, 21), with patient sources spanning emergency departments, general wards, and intensive care units. The mean age of ADHF patients ranged from 56.7 to 82.0 years, reflecting cohorts of predominantly older adults. The proportion of men varied between 41.0 and 58.0%, while reporting of HF phenotype was less consistent: only five cohorts provided data on HFrEF, ranging from 18.9 to 42.4% (15, 18–21), and ischemic etiology was reported in five cohorts, ranging from 17.2 to 62.0% (16, 18, 19, 21). All studies assessed serum chloride at the time of hospital admission or within the first 24 h, with hypochloremia defined using study-specific cutoffs ranging from < 95 to < 98 mmol/L. Accordingly, 2,422 (14.0%) patients with ADHF had hypochloremia at admission. Mortality counts ranged from 18 to 776 deaths per study. Overall, 1,242 (7.2%) patients died during hospitalization. Adjustment for confounders was heterogeneous: three cohorts (16, 18, 19) reported multivariable-adjusted risk estimates incorporating demographics, HF severity, comorbidities, and laboratory parameters, whereas the remaining five cohorts (15, 17, 20, 21) provided unadjusted estimates only.

**TABLE 1:** Characteristics of the included studies.

Study	Country	Design	Diagnosis	No. of patients with ADHF	Mean age (years)	Men (%)	HFrEF (%)	Ischemic HF (%)	Timing and measurements of serum chloride	Cutoff value for hypochloremia	No. of patients with hypochloremia	No. of patients died during hospitalization	Variables adjusted or matched
Radulović et al. (15)	Croatia	PC	ADHF patients from ED	152	77	48	42.4	NR	At admission	<98 mmol/L	11	18	None
Misumi et al. (16) C1	Japan	PC	ADHF patients from ED	1424	80	56.3	NR	29.8	At admission	<98 mmol/L	134	72	Age, sex, GWTG-HF risk score, and BNP
Misumi et al. (16) C2	Japan	RC	ADHF patients from hospital	1256	77	56.8	NR	25.2	At admission	<98 mmol/L	153	61	None
Zhao et al. (17)	China	PC	ADHF patients from hospital	4966	69	58	NR	NR	At admission	<95 mmol/L	381	207	None
Solela et al. (18)	Ethiopia	RC	ADHF patients admitted to medical wards or ICU	303	56.7	48.5	34.3	17.2	At admission	<96 mmol/L	98	26	Age, sex, NYHA class, SBP, DBP, BUN, Sodium, AST, ALT, QRS prolongation, LVEF, and dyslipidemia
Wei et al. (19)	USA	RC	ADHF patients admitted to ICU	7844	73	53.6	39.2	33.7	Within 24 h of admission	<96 mmol/L	1525	776	Age, sex, MI, COPD, DM, CKD, liver disease, CCI, APSIII, HR, MAP, FBG, BUN, serum calcium, serum potassium, eGFR, white cell count, Hb, platelets, and bicarbonate
Nunez et al. (20)	Spain	PC	ADHF patients from hospital	386	82	41	18.9	NR	Within 24 h of admission	<96 mmol/L	59	18	None
Shcheko chikhin et al. (21)	Russia	RC	ADHF patients from ED	908	71.6	55.1	50.3	62	At admission	<98 mmol/L	61	64	None

PC, prospective cohort; RC, retrospective cohort; ADHF, acute decompensated heart failure; ED, emergency department; HFrEF, heart failure with reduced ejection fraction; NR, not reported; GWTG-HF, Get With The Guidelines–Heart Failure; BNP, B-type natriuretic peptide; ICU, intensive care unit; NYHA, New York Heart Association; SBP, systolic blood pressure; DBP, diastolic blood pressure; BUN, blood urea nitrogen; AST, aspartate aminotransferase; ALT, alanine aminotransferase; LVEF, left ventricular ejection fraction; MI, myocardial infarction; COPD, chronic obstructive pulmonary disease; DM, diabetes mellitus; CKD, chronic kidney disease; CCI, Charlson Comorbidity Index; APSIII, Acute Physiology Score III; HR, heart rate; MAP, mean arterial pressure; FBG, fasting blood glucose; eGFR, estimated glomerular filtration rate; Hb, hemoglobin.

### Study quality evaluation

Study quality was assessed using the NOS (Table 2). Overall, NOS scores ranged from 6 to 9, indicating moderate to high methodological quality across the included evidence. Three cohorts (16, 18, 19) achieved the highest rating of 9 points, reflecting strong representativeness of cohorts, rigorous exposure assessment, robust adjustment for major confounders, and adequate follow-up procedures. Four cohorts (15–17, 20) received 7 points, with limitations primarily related to inadequate control for confounding variables despite otherwise sound selection and outcome assessment domains. One study (21) scored 6 points, mainly due to limited cohort representativeness and lack of adjustment for demographic or clinical confounders.

**TABLE 2:** Study quality evaluation via the Newcastle-Ottawa Scale.

Studies	Representativeness of the exposed cohort	Selection of the non-exposed cohort	Ascertainment of exposure	Outcome not present at baseline	Control for age and sex	Control for other confounding factors	Assessment of outcome	Enough long follow-up duration	Adequacy of follow-up of cohort	Total
Radulović et al. (15)	1	1	1	1	0	0	1	1	1	7
Misumi et al. (16) C1	1	1	1	1	1	1	1	1	1	9
Misumi et al. (16) C2	1	1	1	1	0	0	1	1	1	7
Zhao et al. (17)	1	1	1	1	0	0	1	1	1	7
Solela et al. (18)	1	1	1	1	1	1	1	1	1	9
Wei et al. (19)	1	1	1	1	1	1	1	1	1	9
Nunez et al. (20)	1	1	1	1	0	0	1	1	1	7
Shchekochikhin et al. (21)	0	1	1	1	0	0	1	1	1	6

All studies performed well in defining ADHF populations, clearly ascertaining exposure (serum chloride), and ensuring that outcomes were not present at baseline. Variability in confounder control, representativeness of exposed cohorts, and differences in follow-up contributed to score differences.

### Association between hypochloremia and short-term mortality

**FIGURE 2:**
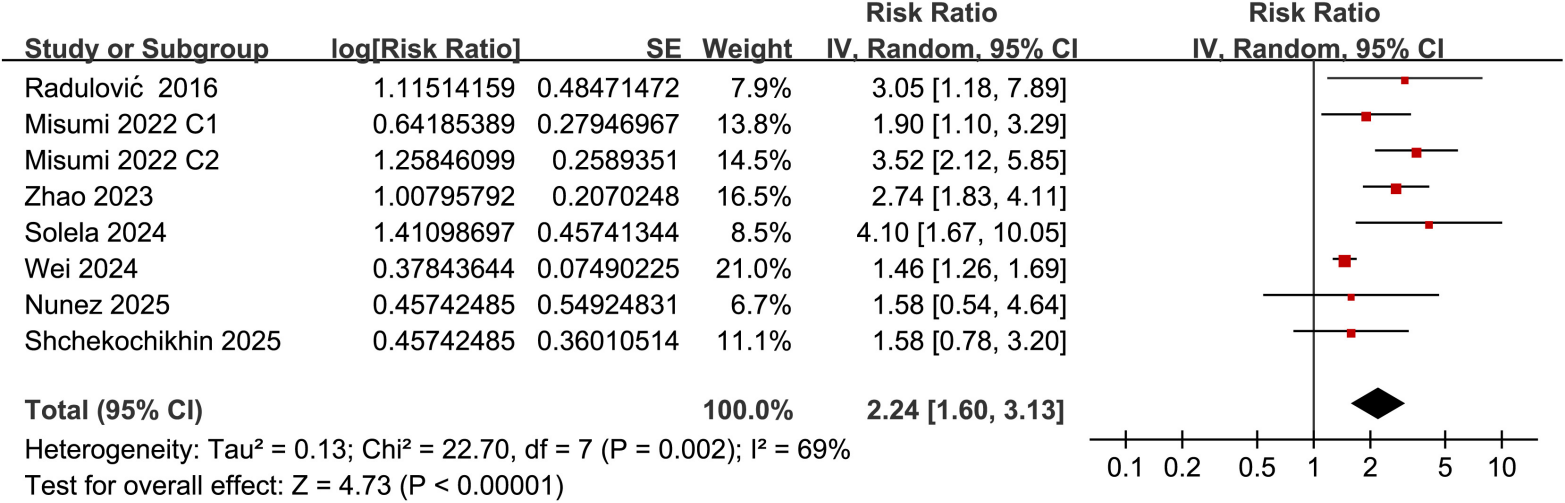
Forest plots for the meta-analysis of the association between hypochloremia and inhospital mortality of patients with ADHF.

**FIGURE 3:**
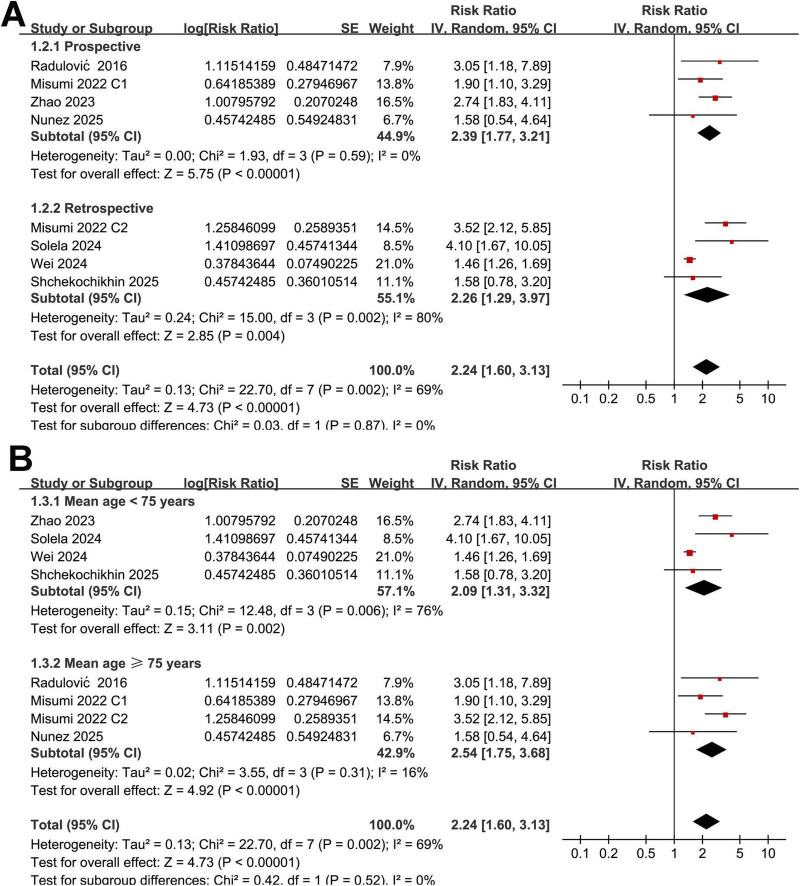
Forest plots for the subgroup analysis of the association between hypochloremia and inhospital mortality of patients with ADHF. **(A)** Subgroup analysis according to study design; and **(B)** subgroup analysis according to mean ages of the patients.

**FIGURE 4:**
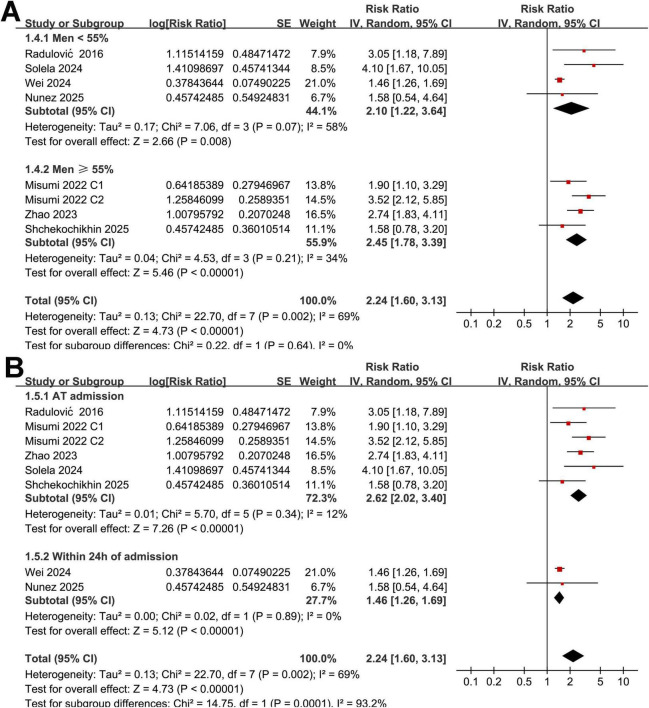
Forest plots for the subgroup analysis of the association between hypochloremia and inhospital mortality of patients with ADHF. (A) Subgroup analysis according to the proportion of men; and **(B)** subgroup analysis according to the timing of serum chloride measurement.

**FIGURE 5:**
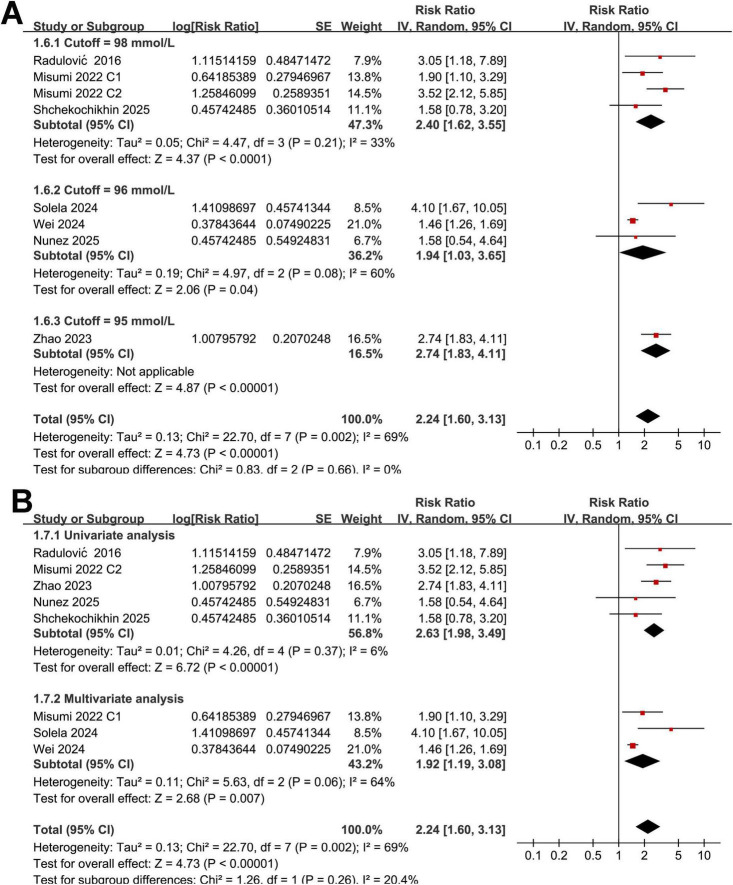
Forest plots for the subgroup analysis of the association between hypochloremia and inhospital mortality of patients with ADHF. **(A)** subgroup analysis according to the cutoff values for hypochloremia; and **(B)** subgroup analysis according to the analytic models.

Pooled results of the eight cohorts from seven studies (15–21) with a random-effects model showed that patients with hypochloremia at baseline were associated with a higher risk of inhospital mortality (RR: 2.24, 95% CI: 1.60–3.13, *p* < 0.001) with moderate heterogeneity (*p* for Cochrane Q test = 0.002; *I*^2^ = 69%; Figure 2). Sensitivity analysis by excluding one study at a time did not significantly change the results (RR: 2.04–2.56, *p* all < 0.05). Further subgroup analysis showed similar results between prospective and retrospective cohorts (RR: 2.39 vs. 2.26, *p* for subgroup difference = 0.87; Figure 3A), in studies with the mean ages of the patients < 75 and ≥ 75 years (RR: 2.09 vs. 2.54, *p* for subgroup difference = 0.52; Figure 3B), and in studies with the proportions of men < 55% and ≥ 55% (RR: 2.10 vs. 2.45, *p* for subgroup difference = 0.64; Figure 4A). Interestingly, a stronger association was observed in studies with serum chloride measured at admission (RR: 2.62, 95% CI: 2.02–3.40, *p* < 0.001; *I*^2^ = 12%) as compared to those with serum chloride measured within 24 h of admission (RR: 1.46, 95% CI: 1.26–1.69, *p* < 0.001; *I*^2^ = 0%; *p* for subgroup difference < 0.001; Figure 4B). Further subgroup analysis showed similar results in studies with the cutoffs for defining hypochloremia of serum chloride < 98, 96, or 95 mmol/L (RR: 2.40 vs. 1.94, and 2.74, *p* for subgroup difference = 0.66; Figure 5A), and in studies with univariate and multivariate analyses (RR: 2.63 vs. 1.92, *p* for subgroup difference = 0.26; Figure 5B).

### Publication bias

**FIGURE 6:**
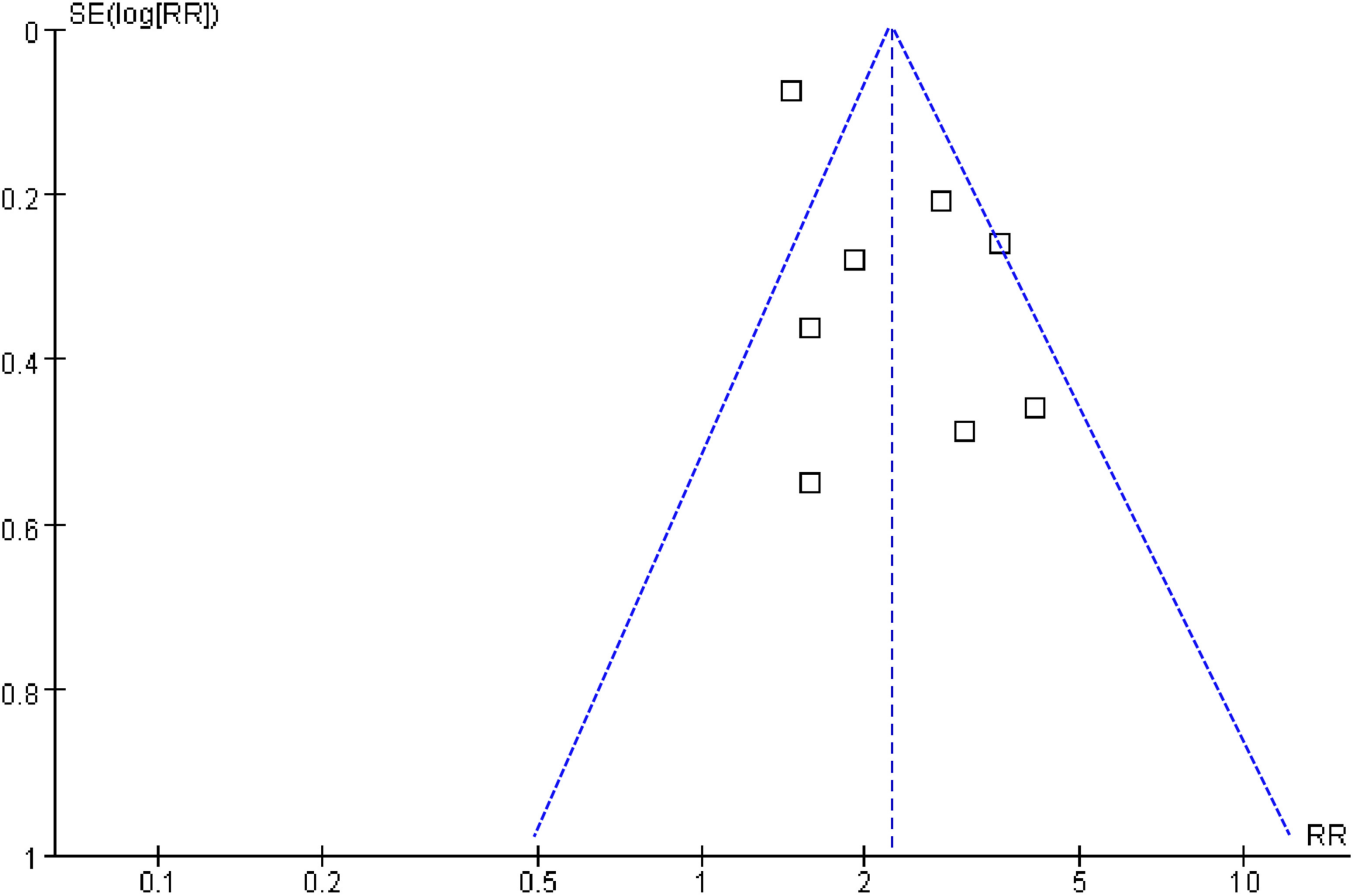
Funnel plots for estimating the potential publication bias underlying the meta-analysis of the association between hypochloremia and inhospital mortality of patients with ADHF.

Figure 6 presents the funnel plots assessing potential publication bias for the meta-analyses of the associations between hypochloremia and short-term mortality of patients with ADHF. The plots appeared symmetrical on visual inspection, indicating a low likelihood of publication bias. This impression was corroborated by Egger’s regression tests, which did not reveal significant asymmetry (*p* = 0.51).

### Certainty of the evidence

According to the GRADE framework, the certainty of evidence for the association between admission hypochloremia and in-hospital mortality in patients with ADHF was rated as low. The certainty was downgraded because all included studies were observational in nature and moderate statistical heterogeneity was observed among studies. Detailed results of the GRADE assessment are shown in Table 3.

**TABLE 3:** GRADE assessment of the certainty of evidence for the association between admission hypochloremia and in-hospital mortality in patients with acute decompensated heart failure.

Outcome	No. of studies (cohorts)	Participants	Study design	Risk of bias	Inconsistency	Indirectness	Imprecision	Publication bias	Effect estimate/Certainty
In-hospital mortality	7 studies (8 cohorts)	17,239	Observational cohort studies	Serious^1^	Serious^2^	Not serious	Not serious	Not detected^3^	RR 2.24 (95% CI 1.60–3.13) Low ⊕⊕○○

^1^The evidence was derived exclusively from observational cohort studies and residual confounding could not be completely excluded despite adjustment in some studies.

^2^Moderate between-study heterogeneity was observed (*I*^2^ = 69%), although subgroup analysis suggested that timing of chloride measurement may partly explain the heterogeneity.

^3^Funnel plot inspection and Egger’s regression test did not indicate significant publication bias. CI, confidence interval; GRADE, Grading of Recommendations Assessment, Development and Evaluation; RR, risk ratio.

## Discussion

In this meta-analysis of eight cohorts including 17,239 patients hospitalized with ADHF, we found that admission hypochloremia was consistently associated with a markedly higher risk of in-hospital mortality. Although individual cohorts differed in sample size, setting, and chloride thresholds, the direction of association was uniform, and the pooled effect size remained robust across multiple sensitivity and subgroup analyses. These findings extend prior work that has primarily focused on long-term outcomes and suggest that low chloride at presentation is not only a marker of chronic risk but also a meaningful indicator of short-term vulnerability during the index hospitalization.

Several mechanisms may explain why hypochloremia portends higher acute mortality in ADHF. Chloride is central to renal tubular handling of sodium and water, and low chloride concentrations can trigger activation of the RAAS system and sympathetic nervous system, thereby promoting vasoconstriction, sodium retention, and persistent congestion (7, 29). Hypochloremia is also strongly linked to loop diuretic exposure and diuretic resistance (30). Paradoxically, more intensive decongestion attempts can lead to further chloride loss, metabolic alkalosis, and impaired response to subsequent diuretic therapy (31, 32). These processes may perpetuate a cycle of refractory volume overload, worsening renal function, and hemodynamic instability—key drivers of early mortality in ADHF (33). Moreover, chloride participates in transmembrane ion transport and acid–base balance (34). Hypochloremic metabolic alkalosis can reduce cerebral and coronary blood flow, predispose to arrhythmias, and impair tissue oxygen delivery, all of which may contribute to adverse outcomes during hospitalization (35).

An important question is whether hypochloremia acts merely as a marker of disease severity or directly contributes to adverse outcomes in ADHF. On one hand, low serum chloride may reflect a more advanced clinical state characterized by severe congestion, neurohormonal activation, renal dysfunction, hemodynamic compromise, and intensive diuretic exposure (36). In this context, hypochloremia may function primarily as an integrated biomarker of high-risk physiology rather than an independent mediator of mortality. On the other hand, accumulating experimental and clinical evidence suggests that chloride itself participates in key regulatory pathways relevant to HF progression. Reduced chloride delivery to the macula densa may stimulate renin release and activation of the renin–angiotensin–aldosterone system, while chloride depletion may contribute to diuretic resistance, impaired natriuresis, metabolic alkalosis, and persistent fluid overload (37). These mechanisms raise the possibility that hypochloremia is not only a marker but may also contribute directly to clinical deterioration. However, because all included studies were observational, residual confounding and reverse causality cannot be excluded. Therefore, the current evidence supports hypochloremia as a clinically useful prognostic marker, whereas its role as a modifiable therapeutic target remains to be established through prospective interventional studies.

The subgroup findings of this meta-analysis provide additional insight into these pathophysiologic links. We observed that the association between hypochloremia and in-hospital mortality was broadly consistent across study designs, age strata, and sex distribution, suggesting that the prognostic relevance of low chloride is not confined to a specific demographic profile or to either prospective or retrospective cohorts alone. Similarly, the association did not materially differ by chloride cutoff (< 98, < 96, or < 95 mmol/L) or by analytic strategy (univariate vs. multivariate models), implying that the relationship between lower chloride and higher risk is graded and relatively robust to differences in modeling. These patterns support the hypothesis that hypochloremia captures a fundamental component of hemodynamic and neurohormonal derangement in ADHF rather than being an artifact of a particular analytic choice or threshold. The most notable heterogeneity emerged when we stratified studies by the timing of serum chloride measurement. The association with in-hospital mortality was stronger when chloride was measured at admission compared with values obtained within 24 h after admission. This temporal pattern is biologically plausible. Admission chloride likely reflects the “unmodified” state of the patient at first medical contact, integrating the cumulative effects of chronic neurohormonal activation, outpatient diuretic therapy, and the acute decompensating trigger. In contrast, chloride levels measured later during hospitalization, even within 24 h of admission, may be influenced by early treatment decisions—such as intravenous diuretics, fluids, or electrolyte supplementation—as well as by hemodilution or partial correction of hypochloremia, thereby attenuating the observed association with mortality (36). From a clinical standpoint, this finding underscores the importance of interpreting chloride values obtained at presentation, before substantial therapeutic interventions have altered the biochemical milieu.

Moderate heterogeneity was observed across the included cohorts, and several clinical and methodological factors may account for this variability. First, patient characteristics differed substantially among studies, including age distributions, prevalence of HFrEF, ischemic etiology, comorbidity burden, and disease severity at presentation. Some cohorts enrolled patients from emergency departments or general medical wards, whereas others included critically ill patients admitted to intensive care units, who generally have higher mortality risks and more profound metabolic disturbances. Second, treatment practices may have varied across healthcare systems and study periods, including differences in diuretic use, fluid management, electrolyte replacement, and implementation of guideline-directed heart failure therapies. Third, the timing of serum chloride measurement varied between studies, with some assessing chloride immediately at admission and others within the first 24 h after hospitalization. Notably, our subgroup analysis identified measurement timing as the only factor significantly associated with differences in effect size, suggesting that early therapeutic interventions may influence the prognostic value of chloride levels. Methodological differences may have also contributed to heterogeneity, including variation in hypochloremia thresholds (<95 to < 98 mmol/L), study design, sample size, and statistical adjustment strategies. Although subgroup analyses generally demonstrated consistent associations across these factors, the combined influence of these clinical and methodological differences likely contributed to the residual heterogeneity observed in the overall analysis.

The strengths of this meta-analysis include a comprehensive and up-to-date literature search, the restriction to longitudinal cohort studies, and the focus on a clinically homogeneous population of patients hospitalized with ADHF and in-hospital mortality as the primary endpoint. We included cohorts from diverse geographic regions and care settings, enhancing the external validity of our findings. Methodological rigor was supported by generally moderate-to-high study quality according to the NOS, as well as by extensive prespecified subgroup and sensitivity analyses that yielded largely consistent results. Leave-one-out analyses showed that no single cohort disproportionately drove the pooled association, and formal testing did not suggest substantial publication bias, which reinforces the robustness of the overall signal.

Several limitations warrant consideration when interpreting these results. First, a substantial proportion of the included studies were retrospective, which may introduce selection bias, misclassification of exposure or outcome, and residual confounding (38). Second, we observed moderate heterogeneity in the magnitude of the association, which likely reflected a combination of clinical and methodological differences among studies, including variations in patient severity, healthcare settings, prevalence of HFrEF and comorbidities, timing of chloride measurement, hypochloremia definitions, treatment strategies, and covariate adjustment models. These sources of variability could not be fully explored because individual patient data were not available. Third, residual confounding remains an important limitation. Although several studies reported multivariable-adjusted estimates, the adjustment strategies varied substantially across cohorts, and many potentially relevant confounders were not consistently reported or controlled for. In particular, markers of congestion severity, such as volume overload, elevated filling pressures, and natriuretic peptide levels, may be associated with both lower serum chloride concentrations and a higher risk of death (39). Similarly, the intensity of diuretic therapy and fluid management strategies may influence chloride balance while also reflecting underlying disease severity (40). Renal dysfunction, which is common in ADHF, may affect electrolyte homeostasis, diuretic responsiveness, and prognosis simultaneously. In addition, concomitant electrolyte abnormalities, including hyponatremia, hypokalemia, and acid–base disturbances, frequently coexist with hypochloremia and may contribute independently to adverse outcomes (41). Because these factors were not uniformly measured or adjusted for across the included studies, their potential influence on the observed association cannot be fully excluded. Accordingly, the findings should be interpreted as evidence of a robust prognostic association rather than proof of an independent causal effect of hypochloremia. Fourth, the observational design precludes definitive causal inference. Although several biologically plausible mechanisms suggest that chloride depletion may contribute directly to adverse outcomes, hypochloremia may also represent a surrogate marker of disease severity, congestion burden, renal dysfunction, neurohormonal activation, or intensive diuretic therapy (36, 42). Consequently, the observed association should not be interpreted as proof of causality, and interventional studies are needed to determine whether correction of hypochloremia can improve clinical outcomes. In addition, according to the GRADE framework, the overall certainty of evidence was low, mainly because the available evidence was derived from observational studies and moderate heterogeneity existed across cohorts. Finally, the number of available cohorts remains modest, and most originated from single-center or regional registries, which may limit the generalizability of our findings. In addition, our search strategy was restricted to peer-reviewed full-text articles published in English and did not systematically include gray literature, conference proceedings, clinical trial registries, or unpublished studies. Consequently, language bias and publication bias cannot be completely excluded, and potentially relevant studies published in other languages or not formally published may have been missed. Although no significant publication bias was detected by funnel plot inspection or Egger’s test, these methods have limited power when only a small number of studies are available.

Despite these limitations, the present findings have important clinical implications. Because serum chloride is routinely measured as part of standard laboratory panels, hypochloremia can be readily incorporated into bedside risk assessment without additional cost or complexity. Our findings suggest that low admission chloride may help identify patients at increased risk of in-hospital mortality who could benefit from closer monitoring and more individualized management during hospitalization (43). Serum chloride should also be interpreted within the broader clinical context, alongside established prognostic indicators such as natriuretic peptides, serum sodium, and markers of renal function. Because the included studies did not consistently evaluate the incremental prognostic value of chloride beyond these established biomarkers, the extent to which serum chloride provides independent or additive prognostic information remains uncertain. Future studies should investigate whether incorporation of chloride into existing risk stratification models improves prognostic performance in patients with ADHF. In particular, prospective cohort studies with comprehensive adjustment for congestion severity, renal function, diuretic exposure, fluid management, and concomitant electrolyte abnormalities are needed to better define the independent prognostic role of serum chloride. Randomized interventional trials evaluating chloride-preserving or chloride-correcting strategies, such as tailored diuretic regimens or targeted electrolyte management, are also warranted to determine whether modifying chloride status can improve clinical outcomes in patients with ADHF.

## Conclusion

In conclusion, this meta-analysis demonstrates that admission hypochloremia is associated with higher in-hospital mortality in patients with ADHF, independent of study design, patient age, sex distribution, chloride cutoff, and analytic strategy, with the strongest association observed when chloride is measured at admission. While causality cannot be established, these findings support serum chloride as a simple and informative biomarker for early risk stratification in ADHF and highlight the need for further research into chloride-centered diagnostic and therapeutic strategies.

## Data Availability

The original contributions presented in this study are included in this article/Supplementary material, further inquiries can be directed to the corresponding author.
