# Supplementary material for: Low serum chloride concentration and the in-hospital mortality of patients with acute decompensated heart failure: a meta-analysis
Source: Front Med (Lausanne). 2026 Jul 10;13:1826977. doi: 10.3389/fmed.2026.1826977 (PMC13395917; doi:10.3389/fmed.2026.1826977)
Supplement: Supplementary file 1 [file Table_1.docx]

**Detailed search strategy for each database**

**PubMed**

#1 Chloride Terms

("Chlorides"[Mesh] OR chloride[tiab] OR "chloride level"[tiab] OR "serum chloride"[tiab] OR hypochloremia[tiab] OR hypochloraemia[tiab])

#2 Heart Failure Terms

("Heart Failure"[Mesh] OR "Heart Failure, Congestive"[Mesh] OR "acute decompensated heart failure"[tiab] OR ADHF[tiab] OR "acute heart failure"[tiab] OR "acute HF"[tiab] OR "decompensated heart failure"[tiab] OR "decompensated HF"[tiab] OR "heart failure"[tiab])

#3 Mortality Terms

("Mortality"[Mesh] OR mortality[tiab] OR death[tiab] OR deaths[tiab] OR survival[tiab] OR "all-cause mortality"[tiab] OR "overall survival"[tiab])

#4 Combine

#1 AND #2 AND #3

#5 Limits

Humans[mesh]

AND ("1900/01/01"[dp] : "2025/10/29"[dp])

**Embase**

1. 'chloride'/exp OR chloride:ti,ab OR 'chloride level':ti,ab OR 'serum chloride':ti,ab OR hypochloremia:ti,ab OR hypochloraemia:ti,ab

2. 'heart failure'/exp OR 'acute heart failure':ti,ab OR 'acute decompensated heart failure':ti,ab OR ADHF:ti,ab OR 'acute HF':ti,ab OR 'decompensated heart failure':ti,ab OR 'decompensated HF':ti,ab OR 'heart failure':ti,ab

3. 'mortality'/exp OR mortality:ti,ab OR death:ti,ab OR deaths:ti,ab OR survival:ti,ab OR 'all-cause mortality':ti,ab OR 'overall survival':ti,ab

4. 1 AND 2 AND 3

5. limit 4 to human

6. limit 5 to clinical study

7. limit 6 to yr="1900 - 2025"

**Web of Science**

TS = (("chloride" OR "chloride level" OR "serum chloride" OR hypochloremia OR hypochloraemia) AND ("acute decompensated heart failure" OR ADHF OR "acute heart failure" OR "acute HF" OR "decompensated heart failure" OR "decompensated HF" OR "heart failure") AND (mortality OR death OR deaths OR survival OR "all-cause mortality" OR "overall survival"))

Document Types: Article, Clinical Trial, Observational Study

Timespan: 1900–2025 (then manually state search ended 29 Oct 2025)
